# Primate ventral striatum maintains neural representations of the value of previously rewarded objects for habitual seeking

**DOI:** 10.1038/s41467-021-22335-5

**Published:** 2021-04-08

**Authors:** Joonyoung Kang, Hyeji Kim, Seong Hwan Hwang, Minjun Han, Sue-Hyun Lee, Hyoung F. Kim

**Affiliations:** 1 Department of Bio and Brain Engineering, College of Engineering, Korea Advanced Institute of Science and Technology (KAIST), Daejeon, 34141 Republic of Korea; 2 Program of Brain and Cognitive Engineering, College of Engineering, Korea Advanced Institute of Science and Technology (KAIST), Daejeon, 34141 Republic of Korea; 3 Center for Neuroscience Imaging Research, Institute for Basic Science, Suwon, Republic of Korea; 4 School of Biological Sciences, Seoul National University (SNU), Seoul, 08826 Republic of Korea

**Keywords:** Consciousness, Decision

## Abstract

The ventral striatum (VS) is considered a key region that flexibly updates recent changes in reward values for habit learning. However, this update process may not serve to maintain learned habitual behaviors, which are insensitive to value changes. Here, using fMRI in humans and single-unit electrophysiology in macaque monkeys we report another role of the primate VS: that the value memory subserving habitual seeking is stably maintained in the VS. Days after object-value associative learning, human and monkey VS continue to show increased responses to previously rewarded objects, even when no immediate reward outcomes are expected. The similarity of neural response patterns to each rewarded object increases after learning among participants who display habitual seeking. Our data show that long-term memory of high-valued objects is retained as a single representation in the VS and may be utilized to evaluate visual stimuli automatically to guide habitual behavior.

## Abstract (web summary)

Ventral striatum is known to be involved in the value update for habit learning. Here, the authors report neural and behavioural correlates for the long-term maintenance of value memory for previously rewarded objects in the ventral striatum of humans and monkeys.

## Introduction

Animals can habitually search for valuable objects after long-term learning. One well-known example is the drug-seeking habit, where animals, including humans, automatically and compulsively search for drugs^1–3^. This habitual seeking behavior is considered to be based on a strong stimulus–response (S–R) association that is insensitive to immediate outcomes, leading to a rapid and automatic response. Moreover, to maximize the reward with limited time and resources, it is critical to generate a habitual seeking response that quickly and accurately detects valuable objects, based on the previously learned values of stimuli^4–6^. How then is the information of sensory input quickly and automatically evaluated to direct the seeking response? One hypothesis is that the brain areas that control the seeking response contain the memory for previously experienced values of sensory stimuli.

The ventral striatum (VS) in the basal ganglia is one of the more likely structures for processing this habitual seeking behavior given its anatomical connections. It directly receives visual sensory input from the temporal cortex and innervates the motor output structures^7–10^, suggesting that it plays a role in connecting visual information directly to motor output. In addition, dopaminergic projection from the midbrain regions to the VS indicates that this area is anatomically suitable for associating visual stimuli with value information^7,11,12^. Thus, the VS is one of the most promising candidate structures in the basal ganglia for the maintenance of value memory underlying habitual seeking. However, previous studies have mainly focused on a role of the VS encoding expectations of immediate outcomes and updating recent changes in reward values during the learning process of habitual seeking^13–16^. It has yet to be determined whether the VS contains memory for object values experienced in the past, even in the absence of any direct outcome.

To investigate whether the VS processes the memory of object values for habitual seeking, we used long-term learning and habitual gaze tasks^17–20^ for both human and non-human primate subjects. We investigated whether learned values were retained in the VS while the subjects passively viewed previously learned objects in the absence of any immediate outcome. Neural responses in the VS during the incidental perception of learned objects were examined using fMRI in human subjects. To clarify the responses of individual neurons in the VS, we conducted single-unit recording in the monkey brain.

## Results

### Learning the values of visual objects

**Fig. 1:**
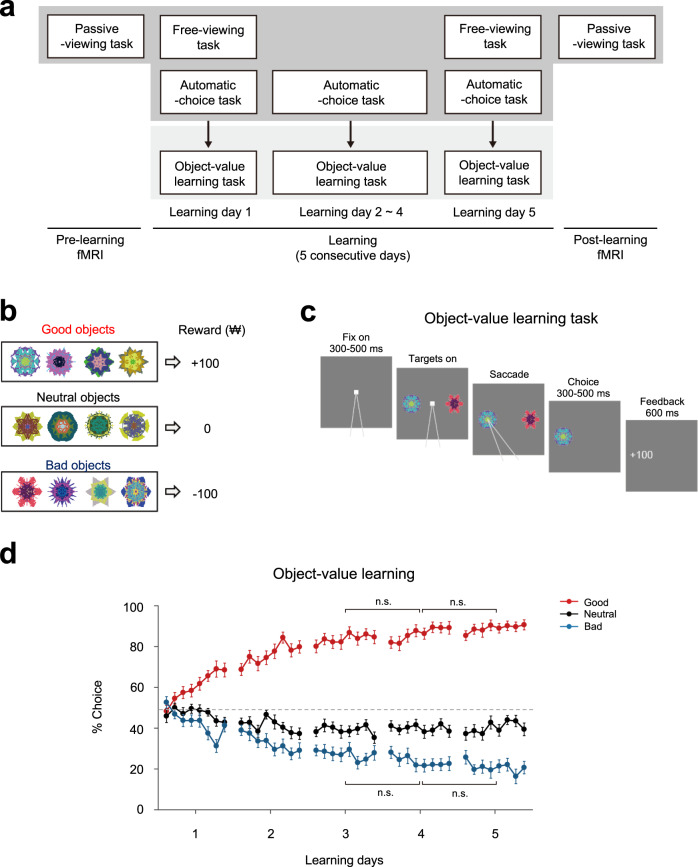
Experimental design and object-value learning in human subjects. **a** The experiment comprised separate Pre-learning, Learning, and Post-learning sessions. The Learning session was conducted for 5 consecutive days, during which the participants learned pairings of 24 fractal objects and values. It was composed of free-viewing, automatic-choice, and object-value learning tasks. On learning days 1 and 5 before the other tasks, the free-viewing task was performed. The object-value learning task was conducted every day of the Learning session. The automatic-choice task was conducted just before the object-value learning task on each learning day. Before and after the Learning session, there were Pre-learning and Post-learning fMRI scan sessions. **b** During the object-value learning task in the Learning session, the participants were trained to associate fractal objects with monetary gain (good; + ₩100), monetary loss (bad; − ₩100), or neither a gain nor a loss (neutral; ₩0). **c** During each trial of the object-value learning task, a white fixation square was followed by the presentation of two fractal objects. The participants were instructed to choose the better of the two objects by making a saccade. The choice phase was followed by a feedback phase showing the outcome (gain, loss, or nothing) earned for that trial. **d** The choice ratio of the objects associated with a good, neutral, or bad value during the object-value learning task. There was an increase of the ratio of choosing good objects as learning proceeded over days during the object-value learning task, while the ratio of choosing neutral or bad objects decreased (*n* = 26 participants, three-way ANOVA with value, day, and bin as factors, ***p = 1.241 × 10^−27^ for value X day; two-way ANOVA with day and bin as factors for the good objects, ***p = 8.956 × 10^−23^ for day; post hoc Bonferroni pairwise comparison, ***p = 5.770 × 10^−9^, between days 1 and 2 for good objects, **p = 4.230 × 10^−4^, between days 2 and 3 for good objects, n.s. not significant). Error bars indicate between-subjects s.e.m.

To test the memory for habitual seeking after long-term learning, our experiment consisted of three sessions, termed the Pre-learning, Learning, and Post-learning sessions (Fig. 1a). The Learning session was conducted in a behavioral testing room outside of the scanner for 5 days. Before the first learning (Pre-learning) and several days after the last learning (Post-learning) sessions, brain activity was monitored using an MRI scanner (Fig. 1a). During the object-value learning task in the Learning session, the participants learned each value of different fractal objects, which were associated with a monetary gain (good; + ₩100), a monetary loss (bad; − ₩100), or neither a gain nor a loss (neutral; ₩0) (Fig. 1b). They were instructed to choose one of two fractal objects by a saccade, and this was followed by the reward feedback (Fig. 1c).

We found a gradual increase of the percentage of choosing good objects as learning proceeded over days during the object-value learning task, while the ratio of choosing neutral or bad objects decreased (three-way ANOVA with value, day, and bin as factors, F(1.378, 34.446) = 132.620, *p* = 5.532 × 10^−15^ for value effect; F(4.400, 110.008) = 58.407, *p* = 1.241 × 10^−27^ for value X day; two-way ANOVA with day and bin as factors for the good and bad objects, respectively, F(2.615, 65.381) = 97.255, *p* = 8.956 × 10^−23^ for day effect of the good objects; F(2.355, 58.878) = 52.534, *p* = 6.263 × 10^−15^ for day effect of the bad objects) (Fig. 1d). A post hoc Bonferroni pairwise comparison revealed significant differences in choice performances between learning days 1 and 2 (*p* = 5.770 × 10^−9^ for good objects, *p* = 4 × 10^−6^ for bad objects), and between learning days 2 and 3 (*p* = 4.230 × 10^−4^ for good objects, *p* = 0.001 for bad objects). The learning performance for choosing good objects showed no difference after 3 days of learning (*p* = 0.287, day 3 vs. 4 and *p* = 0.277, day 4 vs. 5). These results suggest that a 5-day learning period is sufficient to acquire value memory for objects.

### Habitual seeking in human participants

To examine the habitual behavior guided by the previously learned value memory, the participants were asked to perform a free-viewing task, in which they freely viewed previously learned objects without any immediate feedback. This procedure was designed to measure the gazes of the participants without any immediate goal or purpose.

**Fig. 2:**
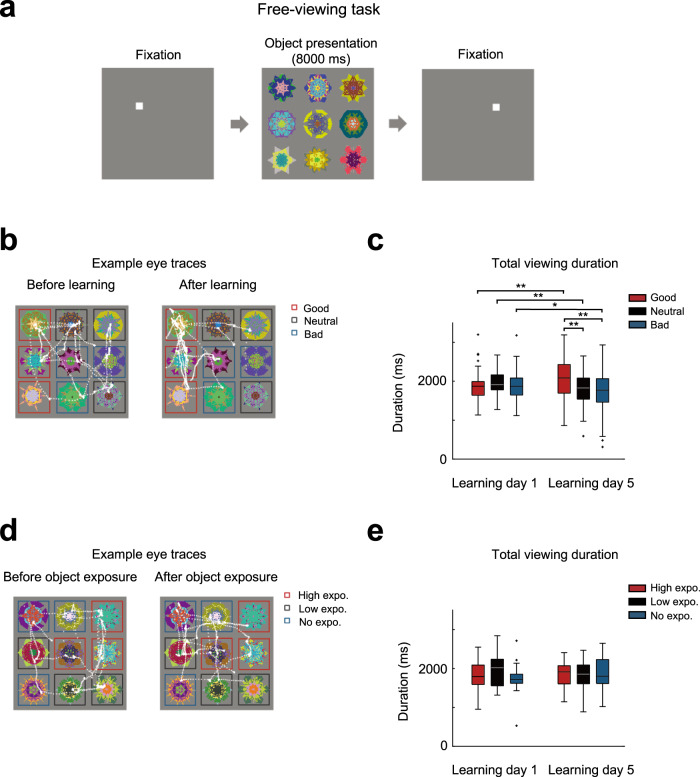
Free-viewing task in human subjects. **a** During each trial of the free-viewing task, a fixation square was followed by the nine objects (three good, three neutral, and three bad objects) presented without any feedback. The participants were instructed freely to observe the nine objects without any feedback. **b** Example eye traces during the free-viewing task before (left) and after (right) object-value learning (learning days 1 and 5). The eye position is depicted by the white dot (2 ms/dots). **c** Boxplots for the total viewing duration for good, neutral, or bad objects during the free-viewing task on learning days 1 and 5. **d** Example eye traces during the free-viewing task before (left) and after (right) exposure to the objects from the control set. **e** Boxplots for the total viewing duration for high-exposure, low-exposure, or no-exposure objects during the free-viewing task for the control set objects. In **c** and **e**, the middle line indicates the median, and the bottom and the top of the box correspond to the 25th and 75th percentiles, respectively. The upper and lower whiskers extend to the highest and lowest values that are within 1.5 times interquartile range of the hinge. Outlying data beyond this are plotted as dots (*n* = 26 participants, two-tailed paired *t* tests, *p < 0.05, **p < 0.01).

In the free-viewing task, nine out of twelve learned objects were pseudorandomly chosen and presented in a 3-by-3 array on the screen for 8 s without any outcomes for each of the trials (Fig. 2a). The eyes of an individual who successfully acquires value memory during 5-day learning may automatically remain longer on fractal objects that were previously associated with good value. Indeed, we found significant differences in the gaze durations for past-learned good, neutral, and bad objects before and after learning (two-way ANOVA, F(2,34.647) = 6.876, *p* = 0.007 for value (good, neutral, bad) X session (pre-learning, post-learning)) (Fig. 2b, c). Accordingly, the gaze duration for good objects increased after learning compared to the duration before learning (*t* = 2.597, *p* = 0.008), while the gaze durations for neutral or bad objects decreased after learning (*t* = 2.899, *p* = 0.004 for neutral objects; *t* = 1.932, *p* = 0.032 for bad objects) (Fig. 2c). Moreover, the gaze duration for good objects was significantly longer than those for neutral or bad objects after learning (*t* = 2.886, *p* = 0.004 for neutral objects; *t* = 2.901, *p* = 0.004 for bad objects) (Fig. 2c). Our data showed that the participants spent more time viewing learned good objects than they did neutral or bad objects, even without a reward outcome. Given the habitual seeking behaviors described in macaque monkey studies^5,18,20^, our data suggest that the participants successfully acquired habitual seeking for good objects.

We then tested the automatic-choice of participants with an automatic-choice task, in which they were instructed freely to choose one of two learned objects without immediate reward feedback, before an object-value learning task on each learning day (Fig. 1a and Supplementary Fig. 1). The ratio of choosing good objects gradually increased over days and was significantly different from the ratio for other value objects (three-way ANOVA with value, day, and bin as factors, F(1.271, 31.770) = 40.889, *p* = 6.211 × 10^−8^ for value effect; F(3.973, 99.336) = 18.988, *p* = 1.403 × 10^−11^ for value X day) (Supplementary Fig. 1). Moreover, during the task on the last day, the ratio of choosing good objects was maintained throughout the repeated exposure to objects without any reward (Supplementary Fig. 1).

It can be argued that a mere exposure effect instead of the learning effect could influence the gaze bias during the free-viewing task after learning^21^, as good objects were viewed more than neutral or bad objects during the value learning procedure. In order to determine if the habitual gaze bias is learning-specific, we conducted an additional control experiment with an independent set of fractal objects. In this experiment, the participants performed a control learning task during which they viewed each object presented on the left or right side of a screen (Supplementary Fig. 2a, b). For this task, the objects were classified into three categories (high, low, and no exposure) according to the exposure time (Supplementary Fig. 2a). We found that the differences in exposure time did not affect the gaze bias in free-viewing task (Fig. 2d, e) or the automatic-choice task (Supplementary Fig. 2c). These results indicate that the gaze bias we observed cannot be accounted for by any difference in the exposure time or familiarity between objects.

### Automatic value discrimination activity in the human VS

**Fig. 3:**
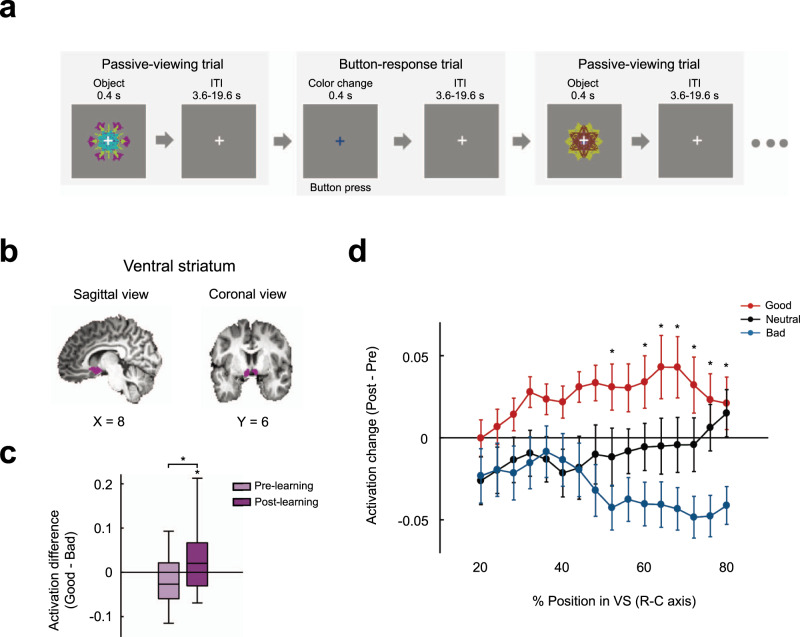
Average magnitude of the BOLD response in the human ventral striatum before and after learning. **a** The passive-viewing task of the Pre-learning or Post-learning fMRI sessions consisted of passive-viewing trial and button-response trial types. On each passive-viewing trial, a fractal object was presented with the white fixation cross, while during each button-response trial, a blue or red fixation cross was presented in the absence of an object. Before the task, participants were instructed to keep their eyes on the fixation cross and to press a button if the white fixation cross changed to blue or red. **b** The ventral striatum (VS, purple) delineated on the MNI brain. **c** Boxplots for the difference in the response magnitude between the good and the bad objects during the Pre-learning and Post-learning sessions. The responses for good objects were significantly greater than those for bad objects after five days of learning (*n* = 22 participants, one-tailed one-sample *t* test against 0, *p = 0.027), but not before learning. Moreover, the activation difference after learning was significantly greater than that before learning (one-tailed paired *t* test, *p = 0.011). The middle line indicates the median, and the bottom and the top of the box correspond to the 25th and 75th percentiles, respectively. The upper and lower whiskers extend to the highest and lowest values that are within 1.5 times interquartile range of the hinge. Outlying data beyond this are plotted as dots. **d** The difference in the response magnitude between the Pre-learning and Post-learning scan sessions for good, neutral, and bad objects at different locations of the VS along the rostral-caudal axis. In the middle and caudal regions, the responses to the good objects were significantly greater than the responses to the bad objects (*n* = 22 participants, one-way ANOVA with value as a factor for each position between the position of 52% of voxels and the caudal end, *p < 0.048; post hoc Bonferroni pairwise comparison, all *p < 0.046 except for the position of 56% of voxels from the rostral end). Error bars indicate between-subjects s.e.m.

To test if the VS processes the value memory, we examined the differences in responses to learned objects while the participants passively viewed each object (passive-viewing task) before and several days (on average 3.4 ± 0.7 days) after the Learning session (Figs. 1a and 3a). To investigate the automatic value discrimination response, we used a human passive-viewing task, which consisted of passive-viewing trials and button-response trials (Fig. 3a). During the passive-viewing trials, object images were presented with a white fixation cross, while the color of the fixation cross changed in the absence of an object image during the button-response trials (Fig. 3a). Throughout the task, the participants were asked to keep their eyes on the fixation cross and to press a button if the color of the fixation cross changed to red or blue. A monetary reward was provided only in the button-response trials. This task was designed to examine incidental or automatic responses to objects with a minimal effect of attention and goal-directed memory retrieval^17,18,20^. The participants showed strong performance during the button-response trials (88.601% ± 13.989% in accuracy). Additionally, in a post-task survey, they reported that they focused on the fixation cross but paid little or no attention to the presented objects. We also found much a greater magnitude of the response during the button-response trials than during the passive-viewing trials before (*t* = 8.983, *p* = 1.223 × 10^−8^) and after (*t* = 5.528, *p* = 1.744 × 10^−5^) learning in the salience network, which is thought to be a higher-order system for the identification for stimuli that are self-relevant with regard to evaluating subjective saliency^22,23^ (Supplementary Fig. 3a). These findings indicate that salience detection for objects was much lower compared to that for the color of the fixation cross.

We examined the BOLD signal changes of the VS for each object during the passive-viewing trials. The average magnitude of responses across all voxels within the VS was calculated (Fig. 3b, c). We found significant differences in the responses between good and bad objects before and after learning (two-way ANOVA, value (good, bad) X session (pre-learning, post-learning): F(1, 21) = 6.215, *p* = 0.021). The neural responses for good objects were significantly greater than those for bad objects after 5 days of learning and a retention period of several days (*t* = 2.048, *p* = 0.027), but not before learning (*t* = 1.105, *p* = 0.141) (Fig. 3c). Moreover, the activation difference after learning was significantly greater than that before learning (*t* = 2.493, *p* = 0.011) (Fig. 3c). This tendency was not observed in the salience network (Supplementary Fig. 3b). Additionally, a whole-brain analysis confirmed that the VS areas showed greater activation for good objects than for bad objects after learning, consistent with the ROI-based analysis results, but no such tendency was found before learning (Supplementary Fig. 4). These results indicate that the activity of the VS reflects the automatic evaluation of objects based on the previously learned value memory, i.e., greater responses to previously learned good objects compared to those to bad objects.

We subsequently analyzed how the value discrimination activity was differentially represented at different locations of the VS along its rostral-caudal axis. Interestingly, the response differences between good and bad objects were noticeable in the middle and caudal regions of the VS, but were relatively weak in the rostral regions (Fig. 3d). In the middle and caudal regions, the responses to the good objects were significantly greater than the responses to the bad objects (one-way ANOVA with value as a factor for each position between the position of 52% of voxels and the caudal end, all F(2, 42) > 3.258, *p* < 0.048; post hoc Bonferroni pairwise comparison, all *p* < 0.046 except for the position of 56% of voxels from the rostral end). These results suggest that the value discrimination responses were prominent in the caudal region of the human VS.

### Direct encoding of value memory in neurons of the monkey VS

The fMRI study showed a BOLD response in the VS that represented the previously learned values of objects. However, the fMRI method has general limitations in elucidating the origin of value memory responses; this then raises the following question: do single neurons in the VS directly process value memory? To address this question, we directly recorded the neuronal responses for previously learned objects from the monkey VS while the monkeys performed a passive-viewing task. The monkeys learned different values of objects that were associated with a water reward (good), no outcome (neutral), or an air puff (bad) (Supplementary Figs. 5a, b, 6a, b). Habitual behavior based on the object-reward association was tested using a free-viewing procedure (Supplementary Figs. 5c and 6c); here, four to six objects were chosen randomly and presented on the screen, and the monkey looked at them freely. No reward was delivered during or after the free-viewing. After long-term learning (>4 days) and a retention period of several days, the monkeys showed a habitual gaze bias toward previously learned good objects (Supplementary Figs. 5c and 6c). The duration and number of habitual gaze for the neutral and bad objects did not differ in the free-viewing task where good, neutral, and bad objects were presented at the same time (Supplementary Fig. 5c).

**Fig. 4:**
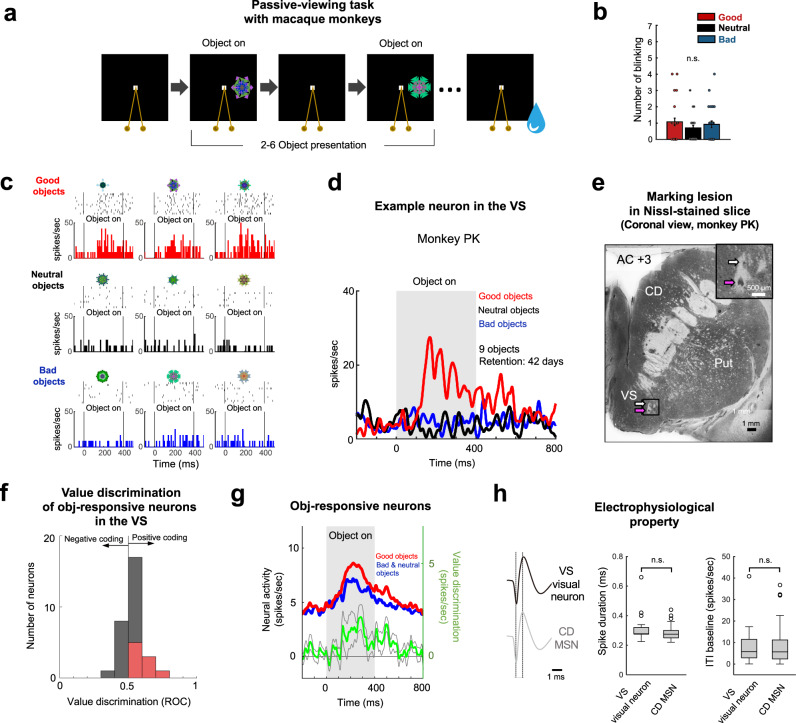
Long-term value memory encoded in neurons of the monkey ventral striatum. **a** Passive-viewing task to examine the neural response to previously learned objects. While the monkey kept fixating at a central white dot, two to six fractal objects were pseudorandomly chosen and sequentially presented. **b** Number of blinking per session of passive-viewing task consisted of 108 trials (*n* = 27 sessions, one-way ANOVA with value as a factor, n.s. not significant). Error bars indicate s.e.m. Individual data points are plotted as dots. **c** An example neuron in the VS that encodes long-term value memory of visual objects. Shown are responses to nine long-term learned objects. **d** Average response of the example neuron to learned objects. Responses to good, neutral, and bad objects are shown in a peristimulus time histogram (PSTH). **e** Location of the example neuron that encodes the long-term value memory in the VS. The marking lesions were located in the VS. The magenta arrow indicates a marking lesion where the example neuron was recorded. Another marking lesion was made 1 mm above the neuron (white arrow). AC anterior commissure. **f** Value discrimination of visual VS neurons (*n* = 30 cells). The difference in response to good and bad or good and neutral objects were calculated as the ROC area. Red bars indicate neurons that showed statistically significant value differences (two-tailed Wilcoxon rank-sum test, *p* < 0.05). **g** Population response of object-responsive neurons in the VS to learned objects in the passive-viewing task. The value discrimination activity is indicated by the green line (good-bad or good-neutral for monkey PK and monkey DN, respectively) (mean ± SEM). **h** Electrophysiological properties of putative medium spiny neurons (MSN) in the caudate nucleus (CD) and object-responsive neurons in the ventral striatum (VS). Object-responsive neurons in the VS (*n* = 30 cells) and MSN (*n* = 43 cells) in the CD had similar spike shapes (left panel), spike durations, and baseline activity levels (two-tailed Wilcoxon rank-sum test, n.s. not significant) (middle and right panels). The middle line indicates the median, and the bottom and the top of the box correspond to the 25th and 75th percentiles, respectively. The upper and lower whiskers extend to the highest and lowest values that are within 1.5 times interquartile range of the hinge. Outlying data beyond this are plotted as dots.

We then examined the value discrimination activity of VS neurons to learned objects during the passive-viewing task (Fig. 4a and Supplementary Fig. 6d). Previously learned fractal objects were tested 21.7 ± 4.4 days and 19.5 ± 5.0 days after the last learning for monkey PK and DN, respectively (mean retention day, mean ± standard error). Both monkeys did not show licking behaviors. Additionally, the blinking responses of monkey PK did not differ between objects regardless of their associated values (Fig. 4b). In the passive-viewing task, previously learned objects were presented sequentially while the monkeys fixated on a central white dot. No outcome was delivered during the object presentation. This task was designed to examine the neural encoding of previously learned values without any effect of a recent outcome or a direct goal associated with fractal objects. Figure 4c shows an example neuron that responded to good objects but not to neutral or bad objects. The average responses of this neuron to learned objects showed remarkable value discrimination activity (Fig. 4d). To identify the recording site, we made an electric marking lesion where the example neuron was recorded. The lesion was located in the VS, confirming that the VS neuron encoded the memory of previously learned values for rewarded objects (long-term value memory) (Fig. 4e and Supplementary Fig. 5d).

Neurons responding to objects in the passive-viewing task (object-responsive neuron) were found more in the monkey VS (8.12%, 16 of 197 neurons for monkey PK; 15.38%, 14 of 91 neurons for monkey DN) than in the caudate head (CDh) which is located above the VS (0.34%, 4 of 1179 neurons for monkey PK; 1.77%, 5 of 282 neurons for monkey DN) (Supplementary Figs. 5d and 6g). Similar to human VS results (Fig. 3d), object-responsive and value-coding neurons were mostly found in the middle and caudal regions of the VS (Supplementary Figs. 5d and 6g). Most object-responsive neurons (70%, 21 out of 30 object-responsive neurons) in the VS positively encoded the previously learned values of visual objects (Fig. 4f), but the value-coding neuron was not found in the CDh, as previously reported^18,24^. Therefore, the population activity of object-responsive neurons in the VS showed a higher response to good objects than to neutral and bad objects (Fig. 4g). However, the neural responses to learned neutral and bad objects did not differ (Supplementary Fig. 7a, e). The electrophysiological property of the visually responsive neurons was not different from the typical type of medium spiny neurons (MSNs) recorded in the caudate nucleus, suggesting that these visually responsive neurons were MSNs in the VS (Fig. 4h). Our single-unit recording data revealed that a population of MSNs in the primate VS directly processed the long-term value memory of visual objects for automatic evaluation. Taken together, our data from both humans and macaque monkeys showed that the value discrimination activity in the VS arose by learning and was stably sustained after learning.

### Better performance in habitual seeking with better long-term value memory

**Fig. 5:**
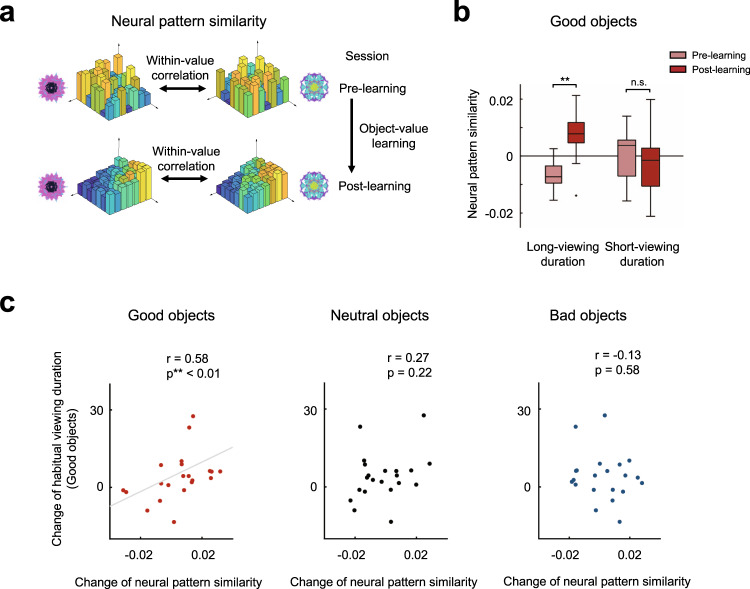
Representations of objects before and after learning in the human ventral striatum. **a** Calculations of neural pattern similarity levels. The within-value correlations were calculated for all possible pairs of good (or neutral or bad) objects, based on the response patterns during the Pre-learning and Post-learning fMRI sessions. **b** Neural pattern similarity during the Pre-learning and Post-learning fMRI sessions for the long-viewing-duration (left) and short-viewing-duration (right) participant groups in the free-viewing task. The neural pattern similarity for good objects increased significantly after learning in the long-viewing-duration group (*n* = 11 participants, two-tailed paired *t* test, **p = 0.002) whereas no change was found in the short-viewing duration group (*n* = 11 participants, two-tailed paired *t* test, *p* = 0.654). n.s. not significant. The middle line indicates the median, and the bottom and the top of the box correspond to the 25th and 75th percentiles, respectively. The upper and lower whiskers extend to the highest and lowest values that are within 1.5 times interquartile range of the hinge. Outlying data beyond this are plotted as dots. **c** Relationship between the performance on the free-viewing task and the neural pattern similarity for good, neutral, or bad objects. The change in the neural pattern similarity for good objects, but not for neutral or bad objects, was significantly correlated with the change of the viewing duration for good objects (*n* = 22 participants, Spearman’s rank correlation, two-tailed, **p = 0.005).

We subsequently investigated whether the neural representations of objects changed after value learning based on the human BOLD response patterns. A multi-voxel pattern analysis (MVPA) was conducted to analyze the value information in the VS, focusing on the BOLD responses for good objects, as learning mainly increased the neural activity for good objects (Figs. 3c and 4f). We derived the degree of neural pattern similarity for good objects by comparing the response patterns of all good objects with each other in the VS (Fig. 5a). The performance of habitual gazing during the free-viewing task may depend on changes in neural representations for good objects.

Notably, the participants with long-viewing durations for good objects showed a significant increase in the similarity of neural response patterns for individual good objects after learning (*t* = 4.224, *p* = 0.002) (Fig. 5b, left). In contrast, the participants with short-viewing durations for good objects showed no significant changes in the similarity of neural response patterns after learning (*t* = 0.462, *p* = 0.654) (Fig. 5b, right). A two-way ANOVA on the similarity of neural response patterns for good objects with sessions (Pre-learning and Post-learning) and duration groups (long and short) as factors revealed significant interaction between the sessions and groups (F(1,20) = 6.69, *p* = 0.018), indicating a different tendency regarding the change of object representation between the long-viewing duration group and short-viewing duration group (Fig. 5b). The increase in the neural pattern similarity was specific to the good objects. No significant changes were found for neutral or bad objects after learning (long-viewing duration group: *t* = 0.437, *p* = 0.671 for neutral objects and *t* = 0.154, *p* = 0.880 for bad objects; short-viewing duration group: *t* = 1.370, *p* = 0.201 for neutral objects and *t* = 0.796, *p* = 0.444 for bad objects). To validate that the increase in the neural pattern similarity for good objects is not simply caused by a broad activity increase, we examined whether voxels arranged in a random order show the same increased similarity effect. By shuffling the order of the voxels and computing the correlations between the randomized voxel responses of good objects, we found that the pattern similarity for good objects after learning in long-viewing duration participants was significantly greater than the similarity derived from the randomized voxel patterns (permutation test, *p* < 0.001). Thus, the VS selectively maintained the object information of positive values that were learned in the past as a single neural representation.

We then investigated the relationship between the individual performance of habitual seeking and the neural pattern similarities for previously learned objects. Consistent with the results from the group analysis (Fig. 5b), the change in the neural pattern similarity for good objects was significantly correlated with the change of the viewing duration for good objects (*r* = 0.575, *p* = 0.005) (Fig. 5c, left panel). However, we did not find significant relationships between the change in the neural pattern similarity for neutral (or bad) objects and the change of the viewing duration for good objects (for neutral objects, *r* = 0.272, *p* = 0.221; for bad objects, *r* = −0.126, *p* = 0.577) (Fig. 5c, middle and right panels). Moreover, we found that the correlation coefficient for good objects was significantly greater than the correlation coefficient for bad objects (*z* = 2.094, *p* = 0.036). These results show that the performance during habitual seeking is selectively dependent on the change in the neural pattern similarity for good objects; participants with greater pattern similarity for good objects show better habitual seeking performance outcomes.

### No long-term value memory in the human CDh

**Fig. 6:**
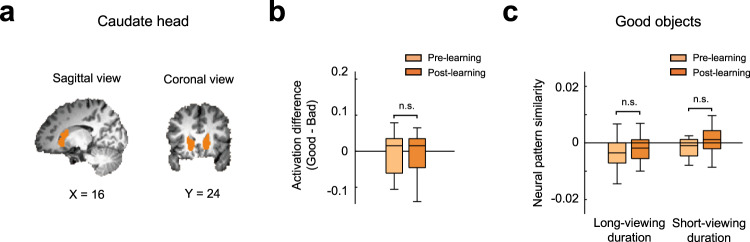
BOLD responses and representational change in the human caudate head. **a** The caudate head (Orange) delineated on the MNI brain. **b** The difference in the response magnitude between the good and the bad objects during the Pre-learning and Post-learning sessions. **c** Neural pattern similarity during the Pre-learning and Post-learning fMRI sessions for the long-viewing-duration (left) and for the short-viewing-duration (right) participant groups in the free-viewing task. n.s. not significant. In **b** and **c**, the middle line indicates the median, and the bottom and the top of the box correspond to the 25th and 75th percentiles, respectively. The upper and lower whiskers extend to the highest and lowest values that are within 1.5 times interquartile range of the hinge. Outlying data beyond this are plotted as dots (*n* = 22 participants, two-tailed paired *t* tests).

Our human and monkey results provide strong evidence that the VS contains long-term value memory for habitual seeking behavior. Because the head region of the caudate nucleus (CDh), placed above the VS, mainly encodes the flexible value of objects but not the long-term value memory^18^, we additionally examined the responses of the CDh as a negative control (Fig. 6a). Unlike the activity in the VS, the CDh did not show differences between the activations in response to good and bad objects after 5-day learning (*t* = 0.109, *p* = 0.914) (Fig. 6b). We also found no significant changes in neural pattern similarity for good objects before and after learning in both the long-viewing and short-viewing duration groups (for long-viewing, *t* = 0.622, *p* = 0.547; for short-viewing, *t* = 1.419, *p* = 0.186) (Fig. 6c). These data suggest that the long-term value memory of objects is processed specifically in the VS, but not in the CDh.

## Discussion

Our data demonstrate that the VS contains long-term memory that underlies evaluations of learned values of stimuli for habitual seeking behavior. We found significant value discrimination responses in both the human and monkey VS after learning and a retention period of several days. Moreover, the similarity of neural representations for good objects increased after learning, an outcome positively correlated with the habitual seeking response for good objects. These findings suggest that the primate VS plays a role in automatic evaluations of objects based on the neural representation of positive values retained since learning, to guide habitual seeking behavior.

### The VS circuit for habitual seeking behavior

How does the VS guide habitual seeking? It is known that neurons in the VS receive visual input directly from the temporal cortex and project this to the motor output structures^8–10,12^. Our data showing that the VS contains value information of learned stimuli combined with its anatomical connection suggest that the information processing in the VS effectively supports the automatic evaluations of visual stimuli and the generation of rapid habitual actions.

Anatomical output from the VS suggests its plausible role in habitual gaze control. Neurons in the VS of macaque monkeys are known to project to motor output structures, such as the substantia nigra pars reticulata (SNr), which directly controls the superior colliculus (SC), the output structure for eye movement^8–10^. In addition, antidromic and orthodromic stimulation in the rat brain confirmed the monosynaptic connection of the VS to the SNr and demonstrated its inhibitory role on the SNr neurons, respectively^25^. These circuit studies suggest how long-term value memory in the VS works for generating a habitual gaze. MSN excitation by the presentation of previously learned good objects in the VS may inhibit SNr neurons through the corresponding inhibitory connection. This inhibition of GABAergic neurons in the SNr (disinhibition) may increase neuronal activity in the SC, eventually facilitating a habitual gaze to good objects. Overall, this simple circuit of the visual cortex-VS-SNr-SC in the primate brain is suitable for generating a fast and automatic habitual seeking response, which is guided by long-term value memory in the VS.

Previous studies showed that neurons in the tail of the caudate nucleus (CDt) encode the value memory for habitual saccades^17,18^. The CDt is a structure that receives inputs directly from neurons in the temporal cortex (visual areas) and from dopamine neurons in the substantia nigra pars compacta (SNc, value-coding region)^24,26^. Thus, it will be important to elucidate how the VS and CDt differentially contribute to habitual seeking. Differences in the anatomical connections provide some clues with which to understand the different roles of the VS and CDt circuits. The main difference was found in the inputs from the limbic system. The VS receives inputs from most of the limbic system including the hippocampus and limbic cortices, whereas the CDt does not^7,27,28^. The VS is thus thought to integrate contextual, emotional, and mnemonic information to control action^28^. The difference in anatomical inputs suggests that the VS may be necessary to combine contextual and emotional information with long-term value memory for guiding habitual behavior.

The VS receives both visual and value inputs from visual and dopamine neurons in the temporal cortex and the midbrain areas, respectively^9,10,12^. Therefore, the VS is anatomically sufficient to associate visual stimuli directly with different values through these visual and dopaminergic inputs. Interestingly, recent studies have shown heterogeneity of the dopamine neurons involved in learning, value memory, and salience^20,29^. CDt-projecting dopamine neurons are known to encode value memory after the initial learning and sustain this memory for more than 1 year^20^. Input from this type of monoaminergic system may provide stability of the value information to the VS. It will be interesting to identify which population of dopamine neurons innervates the VS and to investigate what mechanism generates long-term value memory and how stably this memory is maintained without decay in the VS.

In addition, the brain regions that contain salience-coding neurons, such as the amygdala, orbitofrontal cortex, and SNc, project to the VS^27,29,30^. These anatomical inputs suggest that the VS encodes the salience of learned objects. However, in the passive-viewing task, we did not find salience-coding neurons that responded more strongly to both learned good and bad objects than to neutral objects in the monkey VS (Supplementary Fig. 7). In the fMRI data from the human passive-viewing task, the response of the salience network to learned objects was significantly lower than the response to the fixation cross for reward acquisition (Supplementary Fig. 3a). These results suggest low saliency level during the presentation of learned objects. Additionally, the salience network did not represent long-term value memory (Supplementary Fig. 3b). Therefore, it is likely that the salience of learned objects was little or only slightly reflected in the neural responses of the VS under our task condition, which was designed for showing the memory component. However, it should be noted that our results do not indicate that the VS does not encode salience. It is possible that the response of the salience network mainly reflects the effect of a particular set of tasks such as a spatial attention task. In addition, while no salience-related behavior was observed in the free-viewing task where good, neutral, and bad objects were presented at the same time, our preliminary result showed that the monkey PK showed a gaze bias toward the bad objects when only neutral and bad objects were presented without good objects (number of choice, 4.18 ± 0.07 and 3.99 ± 0.07 for bad and neutral objects, respectively, *p* = 0.047, two-tailed *t* test; gaze duration: 881.35 ± 15.31 ms and 825.25 ± 13.98 ms for bad and neutral objects, respectively, *p* = 0.007, two-tailed *t* test), which is consistent with the previous report^31^. Thus, our monkey data suggest long-term value memory for rewarded and non-rewarded objects in the VS, but the salience process in the VS should be further tested with another task condition for investigating the salience component. Indeed, a previous report showed that the human VS encoded salience in an uncertain reward condition, but not in a certain reward condition^32^.

### Different functions in different subregions of the VS

It has been proposed that different subregions of the basal ganglia structures have different functions^6^. In the macaque monkey brain, the rostral and caudal regions of the basal ganglia process different types of value memory^18–20,33^. Interestingly, our BOLD signals from human participants also showed that long-term memory was more strongly encoded in the caudal VS than in the rostral and medial VS. Indeed, our single-unit recording study showed that more value-coding neurons were found in the caudal region of the monkey VS. These findings suggest functional heterogeneity across the rostral-caudal axis of the primate VS. Our data raise questions about the different roles of neurons in each VS subregion and how these neurons generate these differences. Different anatomical inputs to each VS subregion can generate functional heterogeneity. It will be interesting to study the subcortical and cortical inputs to the rostral and caudal VS in the future.

### Retention of value information in the VS after learning

The most popular conceptualization of the VS role is as a reward-learning center^13,34,35^. Previous rat and human studies proposed dichotomic roles of the dorsal striatum and the VS in controlling action and learning, respectively (actor–critic model)^13,34^. However, recent studies suggest another aspect of the VS role that is related to long-term memory encoding rather than learning^1,28,36–40^. The VS was found to be activated by the presentation of drug-related stimuli in addiction patients, suggesting that neurons in the VS process previously learned experience^1,36,37^. In rat studies, neural activity of the VS increased in response to drug-associative cues or contexts after long-term extinction^38,39^. Moreover, synaptic plasticity associated with drug abuse^41^ and the induction of LTP^42^ in the VS has been reported. The synaptic strength between the VS and the hippocampus was found to be critically related to contextual reward behaviors^43,44^. In addition, molecular mechanisms including the PKM-zeta signaling and the cAMP pathway in the rodent VS were found to maintain long-term memory for seeking behavior^45–47^. Blocking the dopamine receptor in the rat VS after learning immediately impaired the conditioned response to the cue^28,40^. These patient and rodent studies support that the VS is involved in not only learning but also memory processes after learning. In this study, we showed the automaticity of habitual behavior and its stability for several days after the last learning. However, to clarify whether the habitual behavior we observed is a classical habit, it will be necessary to test whether the behavior persists even after a devaluation task, a classical procedure to characterize the habit in the future^48,49^. Additionally, it is possible that BOLD signals from fMRI may be more complex than a direct correspondence with the activity of individual neurons^50–52^. Nevertheless, we found consistent results of the VS response change for subserving long-term value memory in both human fMRI and monkey single-unit recording systems. Our human and macaque monkey data propose a role of the VS as a “director” that guides habitual behavior with the script of value information written in the past.

## Methods

### Part I. Human experiment

#### Participants

Twenty-six neurologically intact right-handed participants (12 females, mean age = 23 ± 3.0 years, range 19–30 years) took part in the experiment. The participants reported that they had normal or corrected-to-normal vision. The data from four of the participants were used only for behavioral analyses because their fMRI acquisition protocol was different from that of the other 22 participants. All participants provided written informed consent for the procedure in accordance with protocols approved by the Institutional Review Board of IBS (Institute for Basic Science), Seoul National University, and KAIST (Korea Advanced Institute of Science and Technology).

#### Stimuli

We used fractal object images created using Fractal Geometry (Supplementary Fig. 8)^53^. The mean luminance was equalized across the images using SHINE (Spectrum, Histogram, and Intensity Normalization and Equalization) toolbox written with Matlab (www.mapageweb.umontreal.ca/gosselif/shine). Twenty-four fractal object images were selected from 72 generated images based on a behavioral attractiveness test taken by a different set of participants (six females, four males). In this attractiveness test, the participants rated the subjective attractiveness of each image on a 1–7 scale (1, most attractive; 7, least attractive), and the 24 images, which scored between 3.6 and 4.6 were used for the experiment. The 24 images were randomly assigned to two object sets (12 fractal objects per set), and for each set, four objects were associated with a monetary gain (good; + ₩100), another four objects were paired with a monetary loss (bad; − ₩100), and the remaining four objects were associated with neither a gain nor a loss (neutral; ₩0) during the Learning session (see Task Design for details). In order to reduce object-specific effects, two different ways of associating each object with the corresponding value were used, and one of these ways was randomly given to each participant.

#### Task design

Our experiment consisted of separate Pre-learning, Learning, and Post-learning sessions. Both the Pre-learning and Post-learning sessions were conducted inside an MRI scanner, while the Learning session was conducted in a behavioral testing room outside of the scanner. The mean interval between the Pre-learning and Learning sessions was 4.6 days (range 2–7 days), and the mean interval between the Learning and the Post-learning sessions was 3.4 days (range 2–5 days). Tasks in the Pre- and Post-learning sessions were created using PsychoPy (www.psychopy.org), and tasks in the learning session were created using BLIP (www.cocila.net/blip).

In the Learning session, the participants were trained to associate each object with a good, neutral, or bad value (Fig. 1b). This session was conducted for 5 consecutive days (learning day 1–5) and was composed of free-viewing, automatic-choice, and object-value learning tasks (Fig. 1a). The free-viewing task was performed on learning days 1 and 5 before the other tasks. During each trial of the free-viewing task, a fixation square was initially presented at one of four possible locations (a visual angle of 5˚ to the left, right, above, and below the central point of the screen) pseudorandomly. After 200–600 ms, nine fractal objects (three good, three neutral, and three bad objects) were presented in a 3-by-3 array for 8 s (Fig. 2a). The participants were instructed freely to observe the nine objects. There were three runs consisting of 16 trials each. The order and location of the objects were randomized and counterbalanced across the runs.

The automatic-choice task was conducted immediately before the object-value learning task on every learning day. The procedure for the automatic-choice task was identical to that of the object-value learning task, except that no outcome or feedback was given and that the position of the initial fixation square was variable (central point, visual angle of 8˚ above and below the central point) (Supplementary Fig. 1a). The location of the initial fixation square was randomized across the trials in order to reduce the potential for motor preparation before each stimulus onset.

The object-value learning task was conducted every day of the Learning session. On each trial of the object-value learning task, a white fixation square was presented first (Fig. 1c). After 300–500 ms, two fractal objects associated with different values were simultaneously presented at visual angle of 10˚ left and right of the central fixation square, respectively. The participants were asked to select one object that was associated with a higher value by making a saccade as rapidly as possible. After they selected an object, that object remained on the screen for 300–500 ms, followed by a feedback phase showing the outcome (gain, loss, or nothing) earned for that trial (Fig. 1c). Every learning day, the participants conducted this object-value learning task for one object set and then for the other object set. For each set, all possible object pairs associated with different values were presented on the screen (96 trials in each set). The order of the object pairs was pseudorandomized.

In the Pre-learning or Post-learning session, the participants performed a passive-viewing task. Each run of the passive-viewing task involved two trial types, a passive-viewing trial and a button-response trial, presented in a fully interleaved event-related fashion (Fig. 3a). There were four runs consisting of 44 trials each per each object set, and the ratio of the passive-viewing trials to button-response trials in a run was 36 to 8. In each of the passive-viewing trials, a fractal object (~7˚ × 7˚) was presented for 400 ms with the white fixation cross. In each of the button-response trials, a blue or red fixation cross was presented for 400 ms in the absence of any object. Between trials, there was a variable inter-trial interval (ITI) of 3.6–19.6 s with an average time of 7.6 s. Before the task, the participants were instructed to keep their eyes on the fixation cross and to press a button if the color of the white fixation cross changed to blue or red. The orders of the different trial types and the different value objects were pseudorandomized and counterbalanced across runs (Fig. 3a).

To examine whether different exposure times to each object can cause gaze bias, we also conducted a follow-up control experiment with a different set of objects (Supplementary Fig. 8). In this experiment, the participants performed the same Learning session but without the object-value learning task. Instead of the object-value learning task, they completed the control learning task. For this task, the 12 fractal objects were classified into high-, low-, and no-exposure categories (Supplementary Fig. 2a). In each trial of the task, one fractal object was presented on the left or right area of the screen. The participants were asked to saccade to the presented object and that object remained on the screen for 300–500 ms (Supplementary Fig. 1b). Each object from the high-exposure category and the low-exposure category was presented 16 times and 8 times, respectively. The objects belonging to the no-exposure category were never presented during the control learning task. Each object appears an equal number of times at the left and right positions of the screen. The order of the objects was pseudorandomized for each participant.

#### Eye tracking data acquisition

During the Learning session, eye-position data of the right eye were acquired with a desktop-mounted Eyelink 1000 eye tracker (SR Research, Mississauga, Ontario, Canada) at a sampling rate of 1000 Hz. The output of the eye tracker was recorded with a data acquisition board (PCIe-6353, National Instruments, USA) interfaced through a shielded I/O connector block (SCB-68, National Instruments, USA). The visual images were presented via a 27-inch monitor (1920 × 1080 resolution, 60 Hz refresh rate).

#### fMRI data acquisition

Participants were scanned on a 3 T MAGNETOM Prisma scanner with a 64-channel Head/Neck coil (Siemens, Erlangen, Germany) at the Center for Neuroscience Imaging Research of the Institute for Basic Science. Whole-brain volumes were acquired using a T2*-weighted echo-planar imaging two-dimensional (EP2D) sequence, with an in-plane resolution of 2.5 × 2.5 mm, and 48 2.5 mm slices (0.25 mm inter-slice gap, repetition time (TR) = 2000 ms, echo time (TE) = 20 ms, matrix size 76 × 76, field of view (FOV) = 192 mm). T1-weighted anatomical scans were acquired at a 1 mm^3^ resolution using the standard MPRAGE (magnetization prepared rapid acquisition gradient echo) sequence. During the Pre-learning or Post-learning scan session, the visual images were viewed via a back-projection display (1024 × 768 resolution, 60 Hz refresh rate) with a uniform gray background.

#### fMRI data analysis

Data analysis was conducted using AFNI (http://afni.nimh.nih.gov), SUMA (AFNI surface mapper), FreeSurfer, and MATLAB scripts. Data preprocessing consisting of slice-time correction and motion correction was conducted.

To derive the BOLD response magnitudes during the passive-viewing task, we used a standard general linear model by means of the AFNI software package (3dDeconvolve using the GAM function). We derived the percent signal change and *t* value of each voxel from the onset of each fractal object presentation (during each passive-viewing trial of the passive-viewing task) or colored cross presentation (during each button-response trial of the passive-viewing task).

For the neural pattern similarity analysis, we used the split-half correlation analysis method^54–56^. The four event-related runs for each object set and each participant were divided into two halves in all possible (three) ways. For each split, the Pearson correlation coefficients between the *t* values associated with each pair of objects from two halves of the data were calculated, and subsequently Fisher *z*-transformed. The neural pattern similarity for good (or neutral or bad) objects for each participant was determined by averaging the *z*-transformed correlation coefficients from all possible pairs of good (or neutral or bad) objects. To examine the relationship between the behavioral performance and the change in the neural pattern similarity, we divided the participants into two groups based on the viewing duration for good objects during the free-viewing task. The participants whose viewing duration for good objects were greater than the median viewing duration across participants were categorized into the long-viewing duration group (*n* = 11), while the others were grouped as the short-viewing duration group (*n* = 11).

#### Regions-of-Interest

The VS was automatically defined by parcellation (“Accumbens-area”) in FreeSurfer. To examine the neural activity at different locations of the VS along its rostral-caudal axis, the VS subregions that corresponded to 40% of the total number of the VS voxels and were centered at every 4% of the VS voxels along the rostral-caudal axis were defined, and the mean signal of each subregion was calculated. The caudate nucleus (CD) was also automatically defined by parcellation (“Caudate”) in FreeSurfer. To define the head region of the CD (CDh), we used the voxels corresponding to the anterior 33% of the length of the CD rostral-caudal^18^. For the salience network, we used preexisting atlases of brain networks (http://findlab.stanford.edu/functional_ROIs.html)^57^. The salience network is an intrinsically connected network anchored in the anterior insula and dorsal anterior cingulate cortex^22,23^.

#### Statistical analysis

To determine statistical significance of the value, day, bin, or interaction effects, repeated-measures ANOVAs (tests of within-subjects effects) were conducted. To examine the detailed effects between factors, the ANOVAs were followed by post hoc Bonferroni comparisons for multiple comparisons or paired *t* tests to compare the means of the two data sets. In the *t* tests, if any interaction effect was revealed in the ANOVA, we used one-tailed *t* tests with the assumption of a predicted direction; otherwise, we used two-tailed *t* tests. For the group comparisons, two-way mixed-model ANOVAs with session (Pre-learning and Post-learning) as a within-subject factor and duration group (long and short) as a between-subject factor were used. For all of the ANOVAs, we used the Greenhouse–Geisser correction if sphericity assumptions were not met. A correlation analysis was done according to Spearman’s rank correlation. To compare the correlation coefficients, we used Fisher’s *r*-to-*z* transformation. To verify the significance of the increase in the neural pattern similarity, a permutation test was performed. For this, we randomly shuffled the order of the voxels and derived the correlations between the randomized voxel responses. We repeated this step 1000 times and tested whether the actual correlation falls within the top 5% of the simulated null distribution of correlations. We used SPSS and MATLAB for statistical analyses.

### Part II. Non-human primate experiment

#### General procedures

Two adult male monkeys (*Macaca mulatta*, 7–9 kg; monkey PK and DN) were used for primate experiments. Animal care and experimental procedures were approved by the Seoul National University and Sungkyunkwan University Institutional Animal Care and Use Committee. Under general anesthesia and surgical conditions, a plastic head holder and a recording chamber were implanted onto the monkeys’ skulls. The position of each chamber was tilted laterally by 25˚ so that it corresponded to the VS. We started the training and recording session after the monkeys were fully recovered from the surgery.

#### Single-unit recording

As a monkey performed a task, the activity of single neurons in the VS was recorded using a general method. The recording sites were determined by means of a 1 mm spacing grid system with the aid of MR images (3 T, Siemens) obtained along the direction of the chamber. Single-unit recording was conducted using a glass-coated electrode (Alpha-Omega). The electrode was inserted into the brain through a stainless-steel guide tube and advanced by an oil-driven micromanipulator (MO-97A, Narishige). The electric signals from the electrode were amplified (with a gain of 10,000; A-M systems Model 3600) and band-pass filtered (0.2–10 kHz; Krohn–Hite Model 3384). Neuronal spikes were isolated online using a custom voltage-time window-discrimination software (BLIP, Laboratory of Sensorimotor Research, National Eye Institute National Institutes of Health [LSR/NEI/NIH], available at www.cocila.net/blip), with the corresponding timings detected at 1 kHz. The waveforms of individual spikes were collected at 50 kHz.

Location of the VS was identified by the recording depth and grid coordination based on the MR images and the brain activity. We mainly confirmed three features to identify the anatomical location of the electrode in the VS of the behaving monkeys: (1) the depth of the first encounter of the striatal neuron below the cortex and white matter, (2) the depth of the first encounter of the VS neuron below the white matter between the caudate and putamen (internal capsule), and (3) the depth of the outside of the brain under the VS. Neuron recording sites were reconstructed on the MR images (3 T, Siemens). To validate the recording sites, an electrical marking lesion was made in the monkey PK (see the electric marking lesion and histology sections).

#### Behavioral tasks

The behavioral procedure was controlled by a Windows-based experimentation data acquisition system (BLIP, LSR/NEI/NIH). The monkey sat in a primate chair, facing a frontoparallel screen in a sound-attenuated and electrically shielded room. Visual stimuli generated by an active matrix liquid crystal display projector (Pro8520HD, ViewSonic) were rear-projected onto the screen. The visual stimuli, created using fractal geometry, were ~8˚ x 8˚ in size. An infrared camera was used to record the eye position and blinking with a sampling rate of 1 kHz (EyeLink 1000, SR Research). Licking was measured by a photobeam system attached to a drink tube (modified from the Coulbourn instruments system).

To examine behavioral and neuronal activities encoding long-term value memory, we conducted two separate phases of procedures: learning (object-value learning task) and memory retrieval (a free-viewing task and a passive-viewing task). The learning was guided by an immediate reward outcome, but the memory retrieval was done with no reward outcome. Details are described below.

#### Object-value learning task

In this learning task, the monkeys learned the values of fractal objects by object-reward association to generate long-term value memory of objects. A set of nine (for Monkey PK) or eight (for Monkey DN) computer-generated fractal objects was used as target objects. While the monkey was fixating on a central white dot, we presented one or two of the objects at right and/or left positions pseudorandomly (10˚ from the center). After 400 ms of fixation dot-object overlap time, the monkey was required to make a saccade to the objects. Each monkey had a slightly different procedure during this learning task: a choice learning task with three object values [good (object was associated with a water reward), neutral (no reward), bad (an air puff)] for monkey PK, and a single-object learning task with two object values [good (water) and neutral (no reward)] for monkey DN. The reward was delivered 600 ms after the monkeys held their gaze on the objects. One training session consisted of 108 trials and 120 trials for monkey PK and monkey DN, respectively. Each set was learned once in 1 day, and the same sets of objects were repeatedly learned across days (>4 days) to generate long-term value memory. Monkeys PK and DN learned total 261 and 312 fractals in total, respectively.

#### Free-viewing task

To examine the behavioral responses of the monkeys to the previously learned objects, we tested free gazes during which no instruction was given while the learned fractal objects were presented. After the monkey fixated on a central white dot for 300 ms, six (for monkey PK) or four (for monkey DN) objects were chosen pseudorandomly and presented simultaneously at symmetric positions (12˚ from the center) (Supplementary Figs. 5c and 6c). The monkey was free to gaze at them for 3 s without any reward outcome. Separated from the free-viewing trials, a reward-associated white dot was presented at one of eight positions during half of the trials to maintain the monkey’s motivation. The reward was delivered when the monkey held its gaze on the white dot for 600 ms.

#### Passive-viewing task

This task was used to examine how the VS neurons responded to the previously learned objects. While the monkey was fixating on a central white dot, two to six objects, selected pseudorandomly from a set of nine (monkey PK) or eight (monkey DN) learned objects, were sequentially presented for 400 ms each at the 15˚ from the center. Each learned object was presented at least eight times in one session. To maintain the monkey’s motivation, the reward was delivered 400 ms after the last object disappeared. The reward was thus not directly associated with any object. The value-coding activity was recorded after long-term learning (>4 days) with a sufficient retention period (>1 day after the last learning session).

#### Electric marking lesion

To reconstruct the location of the VS neurons histologically, we passed a 13 μA negative current for 30 s after the recording of a long-term value memory-coding neuron. The recording site was detected in a Nissl-stained section (Fig. 4e).

Histology monkey PK was deeply anesthetized with an overdose of sodium pentobarbital and perfused transcardially with saline followed by 4% paraformaldehyde. The head was fixed in a stereotaxic frame, and the brain was cut into blocks along the coronal plane including midbrain region. The block was post-fixed overnight at 4 °C, and then cryoprotected for 5 days in increasing gradients of glycerol solutions (5, 10 to 20% glycerol in PBS) before being frozen. The frozen block was cut every 50 µm using a microtome. Every 250-µm-interval slices was underwent for Nissl staining.

### Data analysis

#### Visual response

To examine the neuronal visual responses, we counted the numbers of spikes within the test and control windows for each fractal object in the passive-viewing task (control and test windows: 100–0 ms before object onset and 0–400 ms after object onset, respectively). To test whether the neuron had visual responses, we compared the numbers of spikes between the control and test windows in individual trials for each object. The Wilcoxon rank-sum test was used to test for statistical significance.

#### Value-coding activity

To assess the degree of neuronal discrimination, we initially measured the magnitude of each neuron’s response to each fractal object by counting the numbers of spikes within a test window in individual trials. We analyzed the neural responses during object presentation (0–400 ms time window after object presentation) in the passive-viewing tasks. With regard to neuronal discrimination, we defined it as the area under the receiver operating characteristic (ROC) based on the response magnitudes of the neurons to good objects versus those with other values (neutral and bad). To compare neural responses to each group of objects, the firing rates of individual VS neurons for good versus neutral, good versus bad, and neutral versus bad objects in passive-viewing task were plotted on scatter plots (time window for calculating the firing rate: 0–400 ms after object presentation) (Supplementary Fig. 7c–e). The Wilcoxon rank-sum test was used to test for statistical significance of the neuronal discrimination.

#### Free-viewing behavior

To examine the degree of behavioral discrimination, we measured the gaze duration for each object in the free-viewing task. Each gaze duration was calculated according to the time during which the eye position was positioned on each object.

### Reporting Summary

Further information on research design is available in the Nature Research Reporting Summary linked to this article.

## Supplementary information

Supplementary Information

Reporting Summary

## Data Availability

The data are available from the corresponding authors on reasonable request. Sharing and reuse of data require the expressed written permission of the authors, as well as clearance from the Institutional Review Boards. Source data are provided with this paper.

## Source data

Source Data

## Code availability

The custom codes are available from the corresponding authors on reasonable request. Sharing and reuse of codes require the expressed written permission of the authors.
