# Supplementary material for: Primate ventral striatum maintains neural representations of the value of previously rewarded objects for habitual seeking
Source: Nat Commun. 2021 Apr 8;12:2100. doi: 10.1038/s41467-021-22335-5 (PMC8032767; doi:10.1038/s41467-021-22335-5)
Supplement: Supplementary file 1 — Supplementary Information [file 41467_2021_22335_MOESM1_ESM.pdf]

Supplemental information

Primate ventral striatum maintains neural representations of the value of previously rewarded objects for habitual seeking

Authors
Joonyoung Kang[1,2+], Hyeji Kim[3+], Seong Hwan Hwang [4], Minjun Han[3], Sue-Hyun Lee[1,2*], Hyoung F. Kim[4*]

[1.] Department of Bio and Brain Engineering, College of Engineering, Korea Advanced Institute of Science and Technology (KAIST)
[2.] Program of Brain and Cognitive Engineering, College of Engineering, Korea Advanced Institute of Science and Technology (KAIST), 291, Daehak-Ro, Yuseong-Gu, Daejeon, 34141 Republic of Korea
[3.] Center for Neuroscience Imaging Research, Institute for Basic Science, Suwon 16419, Republic of Korea
[4.] School of Biological Sciences, Seoul National University (SNU), Gwanak-ro, Gwanak-gu, Seoul 08826, Republic of Korea

8 supplementary figures

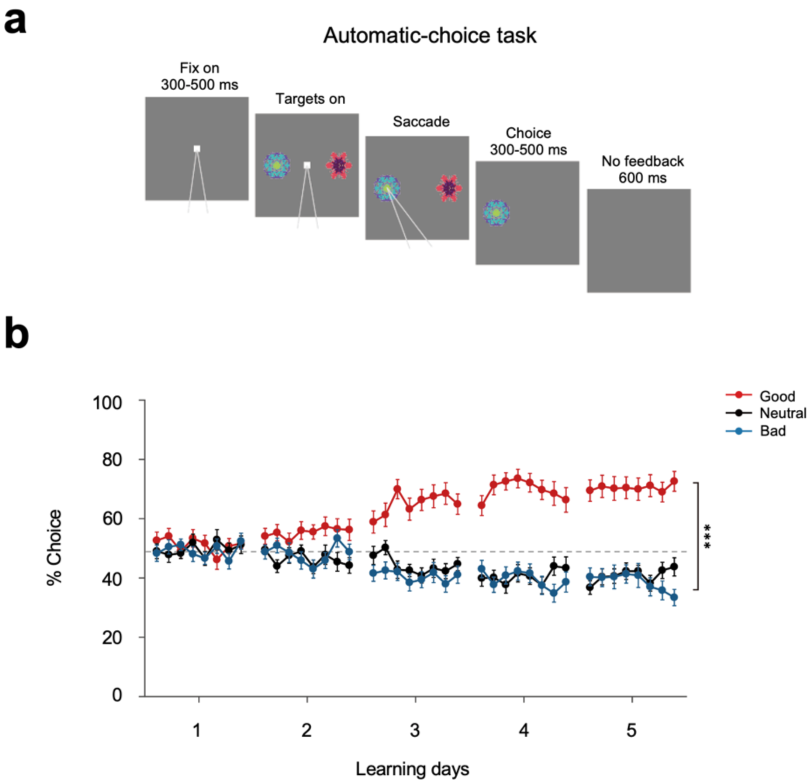

Supplementary Fig. 1. Automatic-choice task in human subjects. **a,** During each trial of the automatic-choice task, a white fixation cross was followed by the presentation of two fractal objects. The participants were instructed to choose one of the objects by making a saccade. There was no feedback. **b,** The choice ratio of the objects associated with a good, neutral, or bad value during the automatic-choice task. There was an increase of the ratio of choosing good objects as learning proceeded over days during the object-value learning task, while the ratio of choosing neutral or bad objects decreased (n = 26 participants, three-way ANOVA with value, day, and bin as factors, ***$p = 6.211 \times 10^{-8}$ for value). Error bars indicate between-subjects s.e.m.

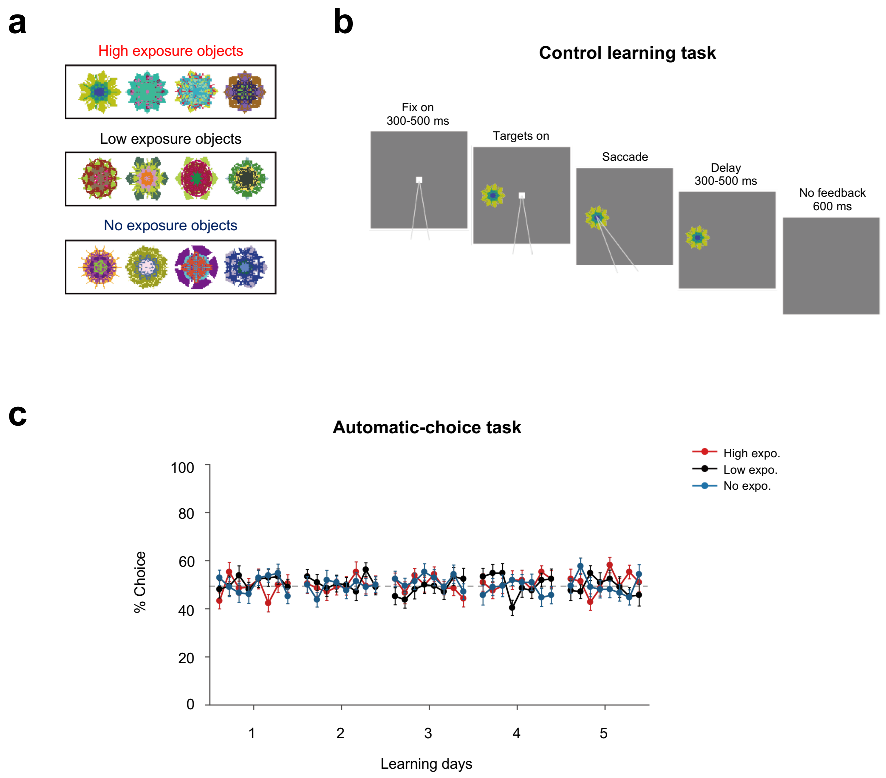

Supplementary Fig. 2. Control learning task in human subjects. **a,** The fractal objects of the control set were classified into high-, low-, and no-exposure categories. **b,** During the control learning task, participants were instructed to view each object presented on the left or right side of the screen. **c,** The ratio of choosing high-, low-, or no-exposure category objects during the automatic-choice task. There was no significant effect of exposure time on the ratio of choosing objects (n = 26 participants). Error bars indicate between-subjects s.e.m.

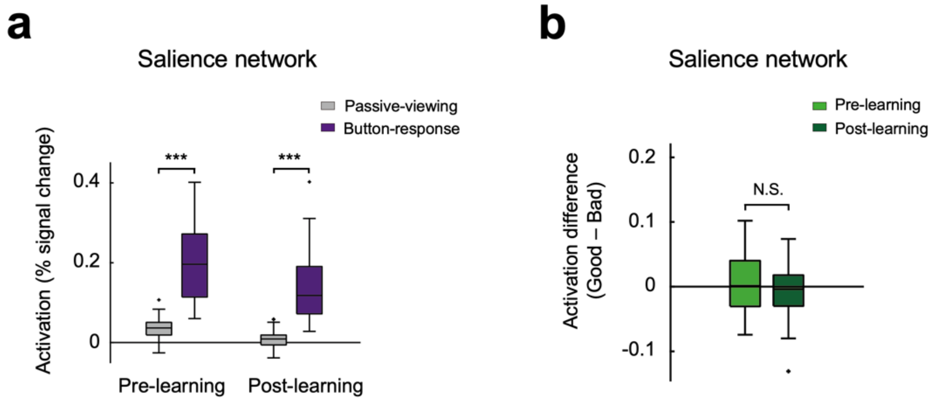

Supplementary Fig. 3. Average magnitude of the BOLD response in the salience network before and after learning in human subjects. **a,** Boxplots for the mean BOLD response for the passive-viewing trials and the button-response trials during Pre-learning and Post-learning sessions. The salience network showed significantly greater responses during the button-response trials in both sessions (***$p = 1.223 \times 10^{-8}$ for Pre-learning, ***$p = 1.744 \times 10^{-5}$ Post-learning sessions). **b,** Boxplots for the difference in the response magnitude between the good and the bad objects during Pre-learning and Post-learning sessions in the salience network. n.s., not significant. In **a** and **b**, the middle line indicates the median, and the bottom and the top of the box correspond to the 25th and 75th percentiles, respectively. The upper and lower whiskers extend to the highest and lowest values that are within 1.5 times interquartile range of the hinge. Outlying data beyond this are plotted as dots (n = 22 participants, two-tailed paired t-tests).

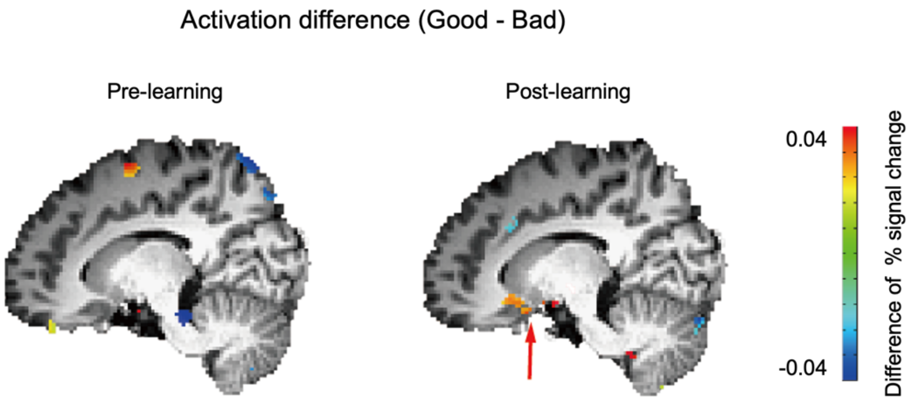

Supplementary Fig. 4. Whole-brain analysis for the activation difference between good and bad objects during the pre-learning (left) and post-learning (right) fMRI sessions. The colored areas show activation differences that are significantly greater than zero across human participants (n = 22 participants, two-tailed t-tests, $p < 0.05$).

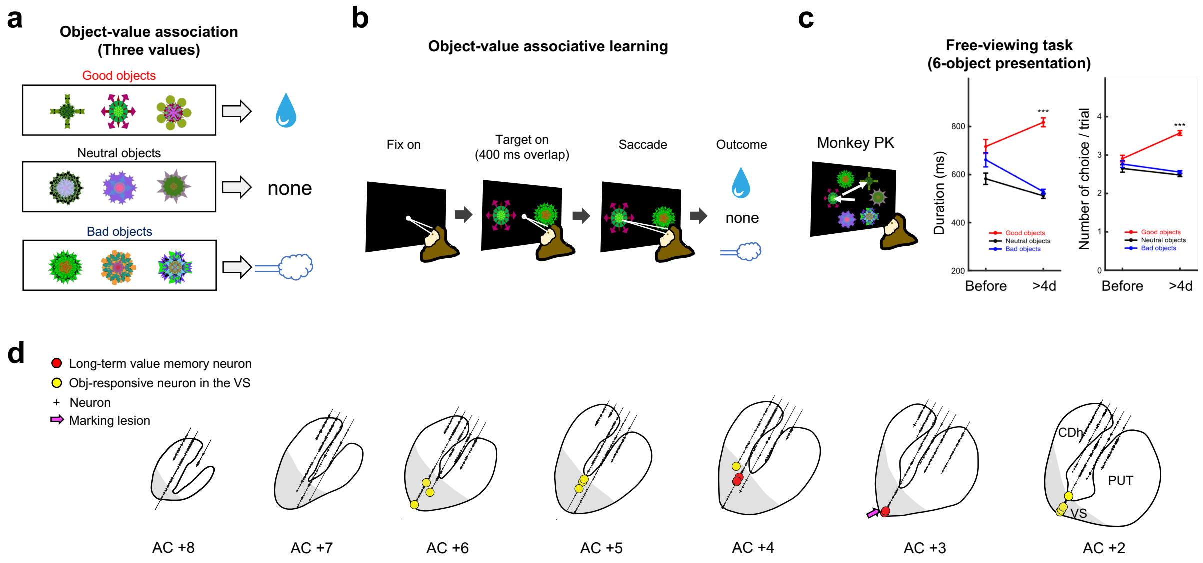

Supplementary Fig. 5. Habitual gaze of monkey PK and the neural recording sites. a, Example set of fractal objects used in the object-value associative learning task and the passive-viewing task. Each set of fractal objects was divided into three groups, good, neutral and bad objects, associated with a liquid reward, no outcome, and an air puff, respectively. **b,** Object-value associative learning task. Two fractal objects were presented at both the right and left positions of a central white dot, and the monkey was required to make a saccade to one of objects. The outcome was delivered after the saccade according to the object-value association. **c,** Free-viewing task for testing habitual gaze to previously learned objects. Six of nine learned objects were simultaneously presented, and the monkey freely looked at it without a direct outcome (left panel). After long-term learning (>4 days) and more than one day of retention, the monkey showed longer gaze duration and greater gaze choice to good objects as compared to neutral and bad objects (mean ± SEM; before: n = 26 sessions, >4d: n = 137 sessions; one-way ANOVA with value as a factor, ***p = 1.484 x 10^{-52} for gaze duration, ***p = 4.303 x 10^{-52} for gaze choice) (middle and right panels). **d,** Locations of visual neurons, value-coding neurons and marking lesion sites. MR-based reconstruction of the recording sites and marking lesions in coronal views of the VS. AC numbers indicate distances from the anterior commissure (AC). CDh: caudate head. PUT: putamen.

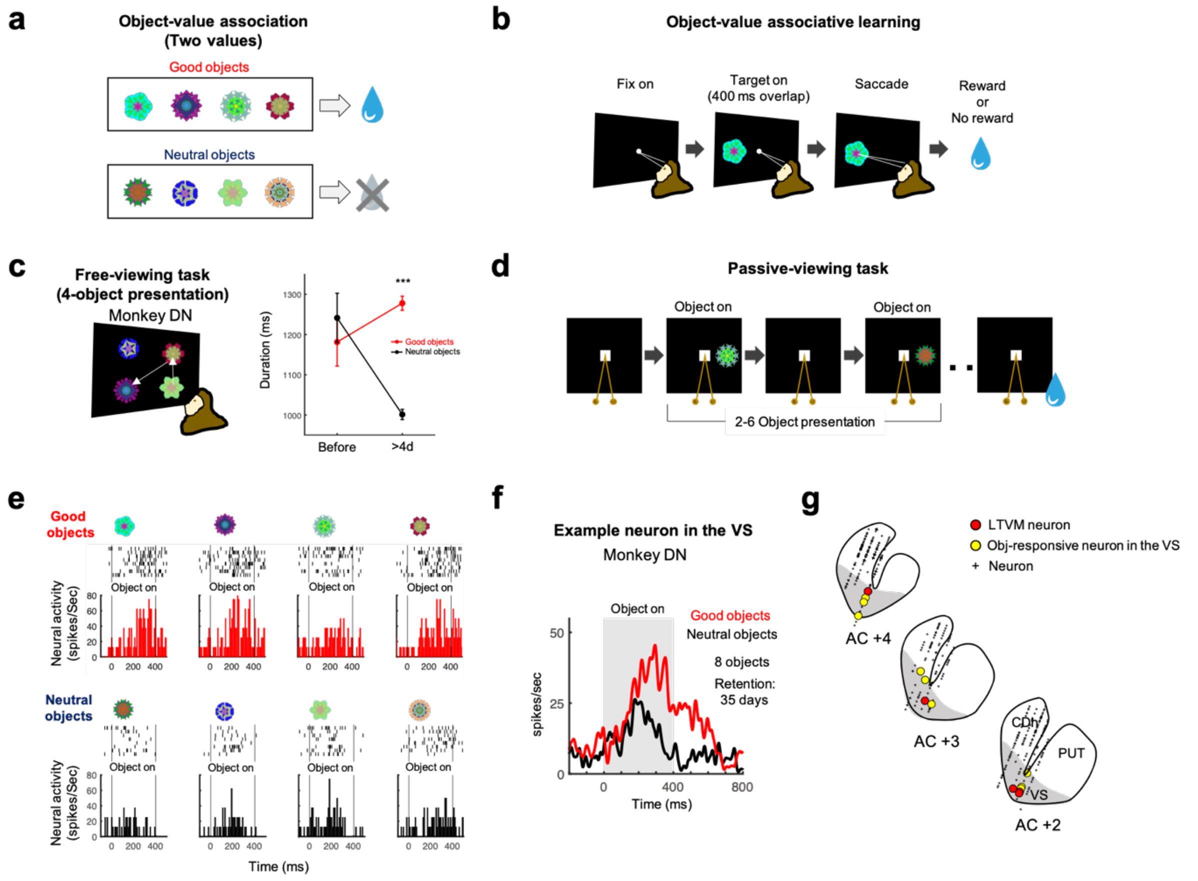

Supplementary Fig. 6. Habitual gaze of monkey DN and long-term value memory neuron in the VS. a, Example set of learned fractal objects used in object-value associative learning task. Two groups of objects were used: Good and neutral objects that were associated with liquid reward and no outcome, respectively. **b,** Object-value associative learning task. The monkey was required to make a saccade to the presented object. **c,** Free-viewing task for testing habitual gaze. Four objects were pseudo-randomly chosen among a set of eight learned objects and simultaneously presented (left panel). The monkey freely looked at it, and showed longer gaze duration to good objects than bad ones (mean ± SEM; before: n = 34 sessions, >4d: n = 281 sessions; two-tailed paired t-tests, ***p = 3.159 x 10^{-34}) (right panel). **d,** Passive-viewing task to test the value-coding activity of the VS neurons. The learned objects were presented sequentially in the neuron's preferred location, while the monkey was fixating at the central white dot. Number of presented objects was randomly decided (2~6). **e-f,** An example neuron that encodes long-term value memory of visual objects. Neural responses to each object (e), and average response of the example neuron to learned objects (f). **g,** Reconstructed recording sites of the visual and value-coding neurons in coronal views. AC numbers indicate distances from the anterior commissure (AC). LTVM neuron: Long-term value memory neuron. CDh: caudate head. PUT: putamen.

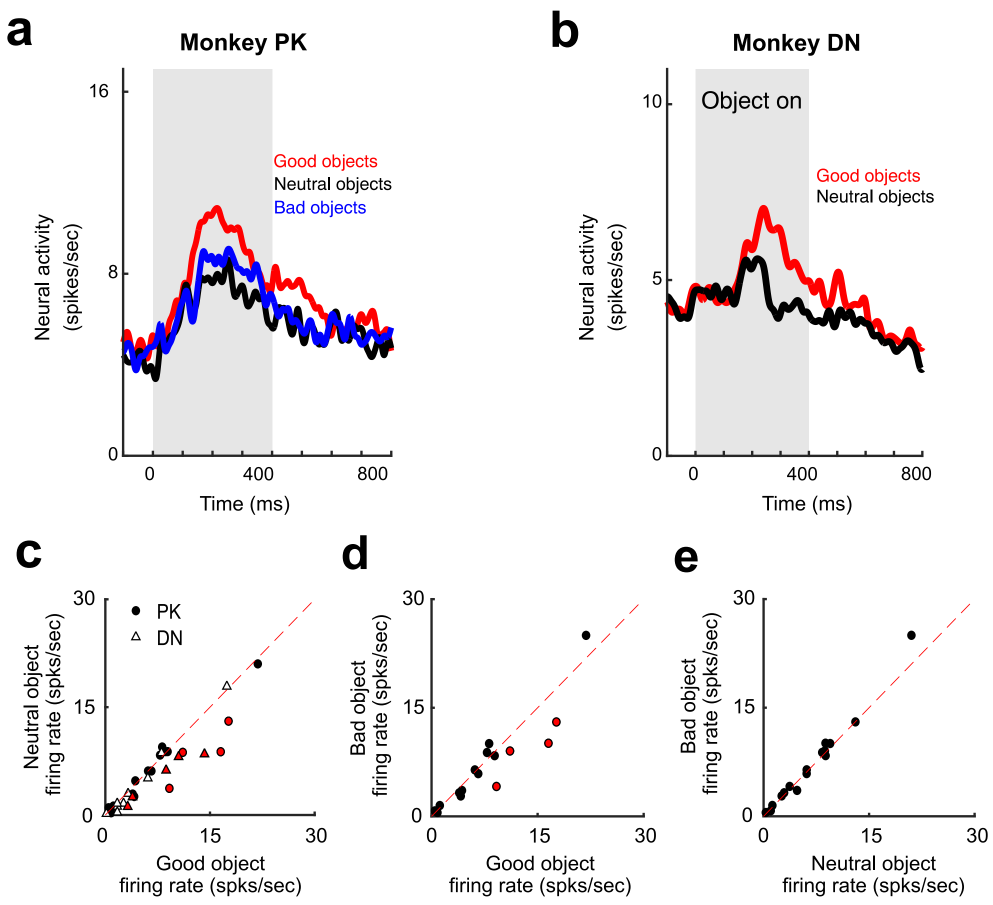

Supplementary Fig. 7. Neuronal responses to previously learned objects in each monkey. a and b, Average responses of object-responsive neurons to previously learned objects in monkey PK **(a)** and monkey DN **(b).** Responses to each object are shown by different colors. Red, black and blue lines indicate the responses to good, neutral and bad objects, respectively. **c-e,** Comparison between neural responses to three types of objects' value. Each object-responsive neuron of two monkeys are plotted with dots (monkey PK, n = 16) and triangles (monkey DN, n = 14). Red-filled circles and triangles indicate neurons with significant value coding (two-tailed Wilcoxon rank-sum test, $p < 0.05$).

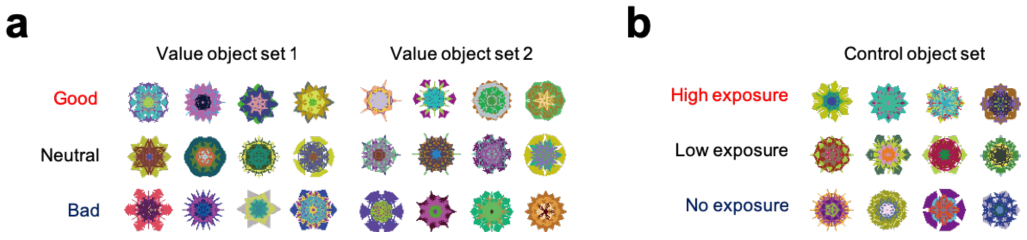

Supplementary Fig. 8. Fractal object images used for object-value learning **(a)** and for control learning **(b)** in human subjects.
