# Supplementary material for: Primate ventral striatum maintains neural representations of the value of previously rewarded objects for habitual seeking
Source: Nat Commun. 2021 Apr 8;12:2100. doi: 10.1038/s41467-021-22335-5 (PMC8032767; doi:10.1038/s41467-021-22335-5)
Supplement: Supplementary file 2 — Reporting Summary [file 41467_2021_22335_MOESM2_ESM.pdf]

nature research

Corresponding author(s): Sue-Hyun Lee and Hyoung F. Kim

Last updated by author(s): Feb 8, 2021

Reporting Summary

Nature Research wishes to improve the reproducibility of the work that we publish. This form provides structure for consistency and transparency in reporting. For further information on Nature Research policies, see our Editorial Policies and the Editorial Policy Checklist.

Statistics

For all statistical analyses, confirm that the following items are present in the figure legend, table legend, main text, or Methods section.

n/a	Confirmed	
☐	☒	The exact sample size (n) for each experimental group/condition, given as a discrete number and unit of measurement
☐	☒	A statement on whether measurements were taken from distinct samples or whether the same sample was measured repeatedly
☐	☒	The statistical test(s) used AND whether they are one- or two-sided<br>*Only common tests should be described solely by name; describe more complex techniques in the Methods section.*
☒	☐	A description of all covariates tested
☐	☒	A description of any assumptions or corrections, such as tests of normality and adjustment for multiple comparisons
☐	☒	A full description of the statistical parameters including central tendency (e.g. means) or other basic estimates (e.g. regression coefficient) AND variation (e.g. standard deviation) or associated estimates of uncertainty (e.g. confidence intervals)
☐	☒	For null hypothesis testing, the test statistic (e.g. F, t, r) with confidence intervals, effect sizes, degrees of freedom and P value noted<br>*Give P values as exact values whenever suitable.*
☒	☐	For Bayesian analysis, information on the choice of priors and Markov chain Monte Carlo settings
☒	☐	For hierarchical and complex designs, identification of the appropriate level for tests and full reporting of outcomes
☐	☒	Estimates of effect sizes (e.g. Cohen's d, Pearson's r), indicating how they were calculated

Our web collection on statistics for biologists contains articles on many of the points above.

Software and code

Policy information about availability of computer code

Data collection	PsychoPy (1.74.0), Fractal Geometry, SHINE toolbox (2010), Siemens MAGNETOM Prisma console, BLIP (1.0)
Data analysis	AFNI (17.2.07), SUMA, Freesurfer (6.0), Matlab (2017a), SPSS (26.0)

For manuscripts utilizing custom algorithms or software that are central to the research but not yet described in published literature, software must be made available to editors and reviewers. We strongly encourage code deposition in a community repository (e.g. GitHub). See the Nature Research guidelines for submitting code & software for further information.

Data

Policy information about availability of data

All manuscripts must include a data availability statement. This statement should provide the following information, where applicable:

- Accession codes, unique identifiers, or web links for publicly available datasets
- A list of figures that have associated raw data
- A description of any restrictions on data availability

The data and custom codes are available from the corresponding authors on reasonable request. Sharing and reuse of data require the expressed written permission of the authors, as well as clearance from the Institutional Review Boards. Source data are provided with this paper.

Field-specific reporting

Life sciences study design

All studies must disclose on these points even when the disclosure is negative.

Sample size	Our main analyses were conducted at the within-subject level in order to demonstrate the effect of long-term learning. The sample size was determined based on recent studies that addressed task-related activation in the striatum using similar analysis methods (e.g., 15 participants in Miller et al., 2014; 16 participants in Robertson et al., 2015; 20 participants in Evers et al., 2017). To collect sufficient data to characterize the presence of long-term value-coding neurons in the monkey VS, we recorded a total of 1749 neurons across brain areas (VS n=288, CDh n=1461) in two monkeys. These sample sizes are typical and sufficient numbers for single unit recording studies (e.g. 484 neurons in White et al., 2019 Nature Communications; 231 neurons in Amita et al., 2020 Nature Communications).
Data exclusions	The data from four of the participants were used only for behavioral analyses and excluded for the fMRI analyses because their fMRI acquisition protocol was different from that of the other 22 participants.<br>No neural data from the monkey VS and CDh were excluded from the analyses.
Replication	In the manuscript, the main conclusion from the human fMRI analyses was replicated at the single cell level using a primate single-unit recording system. Behavioral and neuronal results were consistent with previous studies (Kim and Hikosaka., 2013 Neuron; Kim et al., 2014 Frontiers in Neuroanatomy; Kim et al., 2015 Cell) and among two monkeys in the current study.
Randomization	For our main analyses in the human experimental results, within-subject comparisons were conducted. To examine the relationship between the behavioral performance and the change in the neural pattern similarity, we divided the participants into two groups based on the viewing duration for good objects during the free-viewing task. The participants whose viewing duration for good objects were greater than the median viewing duration across participants were categorized into the long-viewing duration group, while the others were grouped as the short-viewing duration group. To verify the significance of the increase in the neural pattern similarity, a permutation test was performed. For this, we randomly shuffled the order of the voxels and derived the correlations between the randomized voxel responses. We repeated this step 1,000 times and tested whether the actual correlation falls within the top 5% of the simulated null distribution of correlations.<br>No experimental groupings were used for single unit recording study with the monkey VS and CDh.
Blinding	Blinding is irrelevant, as we basically had one group of participants or animals. We divided groups only when we examined the neural responses depending on the level of behavioral performance. In this case we split them up based on the behavioral data.

Reporting for specific materials, systems and methods

We require information from authors about some types of materials, experimental systems and methods used in many studies. Here, indicate whether each material, system or method listed is relevant to your study. If you are not sure if a list item applies to your research, read the appropriate section before selecting a response.

Materials & experimental systems

n/a	Involved in the study	
x		Antibodies
x		Eukaryotic cell lines
x		Palaeontology and archaeology
	x	Animals and other organisms
	x	Human research participants
x		Clinical data
x		Dual use research of concern

Methods

n/a	Involved in the study	
x		ChIP-seq
x		Flow cytometry
	x	MRI-based neuroimaging

Animals and other organisms

Policy information about studies involving animals; ARRIVE guidelines recommended for reporting animal research

Laboratory animals	Adult male monkeys (Macaca mulatta, 6-8 years old, 7-9 kg) were used for primate experiments.
Wild animals	This study did not involve wild animals.
Field-collected samples	This study did not involve samples collected from the field.
Ethics oversight	Animal care and experimental procedures were approved by the Seoul National University and Sungkyunkwan University Institutional Animal Care and Use Committee.

Note that full information on the approval of the study protocol must also be provided in the manuscript.

Human research participants

Policy information about studies involving human research participants

Population characteristics	26 neurologically intact right-handed participants (12 females, mean age = 23 ± 3.0 years, range 19-30 years) took part in the experiment. The participants reported that they had normal or corrected-to-normal vision.
Recruitment	Experiment recruitment flyers were announced via online platforms. There was no potential self-selection bias.
Ethics oversight	All participants provided written informed consent for the procedure in accordance with protocols approved by the Institutional Review Board of IBS (Institute for Basic Science), Seoul National University, and KAIST (Korea Advanced Institute of Science and Technology)

Note that full information on the approval of the study protocol must also be provided in the manuscript.

Magnetic resonance imaging

Experimental design

Design type	task state; event-related design
Design specifications	In the Pre-learning or Post-learning scan session, the participants performed a passive-viewing task. Each run of the passive-viewing task involved two trial types, a passive-viewing trial and a button-response trial, presented in a fully interleaved event-related fashion. There were 4 runs consisting of 44 trials each per each object set (6 min 20 secs for each run), and the ratio of the passive-viewing trials to button-response trials in a run was 36 to 8. In each of the passive-viewing trials, a fractal object was presented for 400 ms with the white fixation cross. In each of the button-response trials, a blue or red fixation cross was presented for 400 ms in the absence of any object. Between trials, there was a variable inter-trial interval (ITI) of 3.6-19.6 s with an average time of 7.6 s.
Behavioral performance measures	During the automatic-choice task or the object-value learning task, the participants were asked to choose one object by making a saccade. We mainly compared the mean ratio of choosing good, neural, or bad objects. During the free-viewing task, the participants were instructed freely to observe the presented nine objects. We mainly measured viewing durations for each object.

Acquisition

Imaging type(s)	functional MRI
Field strength	3T
Sequence & imaging parameters	Whole brain volumes were acquired using a 64-channel Head/Neck coil and a T2*-weighted echo-planar imaging two-dimensional (EP2D) sequence, with an in-plane resolution of 2.5 x 2.5 mm, and 48 2.5 mm slices (0.25 mm inter-slice gap, repetition time (TR) = 2000 ms, echo time (TE) = 20 ms, matrix size 76 x 76, field of view (FOV) = 192 mm). T1-weighted anatomical scans were acquired at a 1 mm^3 resolution using the standard MPRAGE (magnetization prepared rapid acquisition gradient echo) sequence.
Area of acquisition	A whole brain scan was used.

Diffusion MRI ☐ Used ☒ Not used

Preprocessing

Preprocessing software	Data analysis was conducted using AFNI (http://afni.nimh.nih.gov). EPI data were corrected for slice timing differences and head motion.
Normalization	The percent signal change was calculated for each run and each subject on a voxel-by-voxel basis.
Normalization template	Data were not normalized.
Noise and artifact removal	We utilized a standard general linear model with the AFNI software package (3dDeconvolve using the GAM function) to deconvolve the event-related responses. To derive BOLD response magnitudes for each stimulus, the trials of each stimulus (onset time) were modeled as separate events, and participant-specific movement parameters were included in the model as regressors of no interest.
Volume censoring	No volume censoring was performed.

Statistical modeling & inference

Model type and settings	We utilized a standard general linear model with the AFNI software package (3dDeconvolve using the GAM function) to deconvolve the event-related responses. To derive BOLD response magnitudes for each stimulus, the trials of each stimulus (onset time) were modeled as separate events, and participant-specific movement parameters were included in the model as regressors of no interest.

	We used univariate analyses to examine the average magnitude of the responses in each ROI, and also used multivariate analyses to examine the neural pattern similarity in each ROI.
Effect(s) tested	We concluded the result is statistically significant when p-value is less than 0.05. To determine statistical significance of the value, day, bin, or interaction effects, repeated-measures ANOVAs (tests of within-subjects effects) were conducted.

Specify type of analysis: ☐ Whole brain ☒ ROI-based ☐ Both

Anatomical location(s)	The ventral striatum (VS) was automatically defined by parcellation ('Accumbens-area') in FreeSurfer. The caudate nucleus (CD) was also automatically defined by parcellation ('Caudate') in FreeSurfer. For the salience network, we used pre-existing atlases of brain networks (http://findlab.stanford.edu/functional_ROIs.html). The salience network is an intrinsically connected network anchored in the anterior insula and dorsal anterior cingulate cortex.

Statistic type for inference (See Eklund et al. 2016)	Voxel-wise analysis
Correction	For all of the ANOVAs, we used the Greenhouse-Geisser correction if sphericity assumptions were not met. To examine the detailed effects between factors, the ANOVAs were followed by post hoc Bonferroni comparisons for multiple comparisons.

Models & analysis

n/a	Involved in the study
☒	☐ Functional and/or effective connectivity
☒	☐ Graph analysis
☒	☐ Multivariate modeling or predictive analysis
